# Simultaneous synthesis of treatment effects and mapping to a common scale: an alternative to standardisation

**DOI:** 10.1002/jrsm.1130

**Published:** 2015-01-23

**Authors:** AE Ades, Guobing Lu, Sofia Dias, Evan Mayo-Wilson, Daphne Kounali

**Affiliations:** a School of Social and Community Medicine, University of Bristol39 Whatley Road, Bristol, BS8 2PS, UK; b Centre for Outcomes Research and Effectiveness, Research Department of Clinical, Educational and Health Psychology, University College London1-19 Torrington Place, London, WC1E 7HB, UK

**Keywords:** evidence synthesis, multiple outcomes, mapping, social anxiety

## Abstract

**Objective:**

Trials often may report several similar outcomes measured on different test instruments. We explored a method for synthesising treatment effect information both within and between trials and for reporting treatment effects on a common scale as an alternative to standardisation

**Study design:**

We applied a procedure that simultaneously estimates a pooled treatment effect and the “mapping” ratios between the treatment effects on test instruments in a connected network. Standardised and non-standardised treatment effects were compared. The methods were illustrated in a dataset of 22 trials of selective serotonin reuptake inhibitors against placebo for social anxiety disorder, each reporting treatment effects on between one and six of a total nine test instruments.

**Results:**

Ratios of treatment effects on different test instruments varied from trial to trial, with a coefficient of variation of 18% (95% credible interval 11–29%). Standardised effect models fitted the data less well, and standardised treatment effects were estimated with less relative precision than non-standardised effects and with greater relative heterogeneity.

**Conclusion:**

Simultaneous synthesis of treatment effects and mapping to a common scale make fewer assumptions than standardising by dividing effects by the sample standard deviation, allow results to be reported on a common scale, and deliver estimates with superior relative precision. © 2015 The Authors. *Research Synthesis Methods* published by John Wiley & Sons, Ltd.

## 1. Introduction

Standardisation is frequently used in meta-analysis in order to synthesise results from trials that have reported outcomes on similar, but different, measurement scales. The standardised treatment effects are formed by dividing the mean difference between the treatment and control groups by the pooled sample standard deviation (SD; Borenstein *et al*., 2009; Higgins and Green, 2008). The use of standardised effect sizes is particularly prevalent in meta-analyses in psychology and educational and social sciences, but it is also very common in medicine. The claim is that “expressing the effect sizes in SD units makes it possible to compare outcomes across studies” (Light and Pillemer, 1984). The two main approaches are the *d* statistic (Cohen, 1969) in which the mean treatment effect is divided by the pooled SD and the *g* statistic (Hedges, 1981), which includes a small correction for bias because of small sample size, although the correction is very small unless the sample size is less than 10 (Borenstein *et al*., 2009).

Standardisation is attractive because of the proliferation of different test instruments on patient-reported or clinician-reported outcomes such as depression, anxiety, and pain. However, in spite of its popularity, a number of questions can be raised. First, in using the sample SD, the use of Cohen's *d* or Hedge's *g* introduces additional heterogeneity in treatment effects because the sample SDs are subjected to sampling variation, as well as being sensitive to skew and extreme values. Second, even in large trials with minimal sampling variation in the SD, the population SDs may themselves vary considerably from trial to trial. This is partly because treatments may be selectively targeted at different patient populations, for example, at “mild,” “moderate,” or “severe” patients. Some trialists may deliberately limit within-group variation to obtain greater power with a smaller number of patients: This is, after all, no more than sound experimental practice.

For these reasons, leading epidemiologists have been long-standing critics of standardisation in regression (Greenland *et al*., 1986; Greenland *et al*., 1991), and their criticisms apply with equal force to treatment effects in trials. *Modern epidemiology* describes standardised effect sizes as “non-comparable and useless for meta-analysis” (Rothman *et al*., 2008) and notes the possibility of “severe distortions” that “can even reverse the order of strength of results.”

A further seldom mentioned but very strong assumption is that all tests are linear transformations of the same underlying measurement scale, which implies that they are all equally reliable and equally valid and thus equally sensitive to treatments and that correlations between all tests are the same. Therefore, if there was no sampling error, then the ratios of standardised effect sizes within each trial would be unity. This is a hypothesis that can be examined. Other assumptions required by standardisation, but often not met, have been reviewed previously (Grissom and Kim, 2001).

One of the reasons for the enduring attraction of standardisation may be that no fully satisfactory alternative method of synthesising “similar” outcomes both within and between trials has been proposed. Multivariate random-effects meta-analysis (Riley *et al*., 2007; Jackson *et al*., 2011; Wei and Higgins 2013b; Bujkiewicz *et al*., 2013; Mavridis and Salanti, 2012) can be applied to data of this sort, but it does not succeed in expressing the treatment effects on a common scale. A range of heuristic approaches have been suggested, which are examined in more detail in the Discussion section. In this paper, we apply another approach to the synthesis of multiple related outcomes, derived from a structural equation model of psychometric tests (Lu *et al*., 2013; Lu *et al*., 2014). This model assumes that the ratios of the true treatment effects are fixed or may vary slightly between trials. It thus makes a similar but weaker assumption than standardisation, which assumes that the ratios of standardised effects are one. The method simultaneously pools the treatment effects, within and between trials, and maps them onto a common scale. In a previous paper (Lu *et al*., 2014), we applied these models to a multioutcome synthesis of trials on biologic treatments for ankylosing spondylitis. In this paper, we apply the same models to a meta-analysis of pharmacological treatments for social anxiety and use them to compare standardised and non-standardised treatment effects.

## 2. Methods

### 2.1. Illustrative example

As an illustration, we present a synthesis of 22 placebo-controlled trials of selective serotonin re-uptake inhibitors and selective noradrenaline re-uptake inhibitors for social anxiety disorder. These are the trials listed in the National Institute of Health and Care Excellence (NICE) Clinical Guidelines for social anxiety disorders (National Collaborating Centre for Mental Health, 2013), except that we have excluded studies with less than 30 patients per arm and studies in which neither standard errors nor SDs nor ranges were reported on any outcomes. Two trials included an arm with drug plus a non-drug therapy. There were 15 two-arm, 6 three-arm, and 1 four-arm trials, each reporting between one and six out of a total nine different scales. The nine scales were the 144-point Liebowitz Social Anxiety Scale (LSAS; Heimberg *et al*., 1999), the 7-point Clinical Global Impression—Severity (CGI-S; Busner and Targum, 2007), the 72-point Brief Social Phobia Scale (BSPS; Davidson *et al*., 1997), the 40-point Fear Questionnaire—Social Phobia (FQ-SP; Marks and Mathews, 1979), the 30-point Fear of Negative Evaluation (FNE; Watson and Friend, 1969), the 28-point Social Avoidance and Distress Scale (SADS; Watson and Friend, 1969), the 192-point Social Phobia and Anxiety Inventory—Social Phobia (SPAI-SP; Turner *et al*., 1996), the 30-point Sheehan Disability Scale (SDS; Sheehan, 1983), and the 68-point Social Phobia Inventory (SPIN; Connor *et al*., 2000). CGI-S is not specifically intended for use in social anxiety, although the closely related CGI—Improvement has been shown to have similar sensitivity to treatment as other measures of social anxiety (Hedges *et al*., 2009). One trial reported 1 outcome, 4 reported 2, 13 reported 3, 2 reported 4, and 1 each reported 5 and 6.

**Figure 1:**
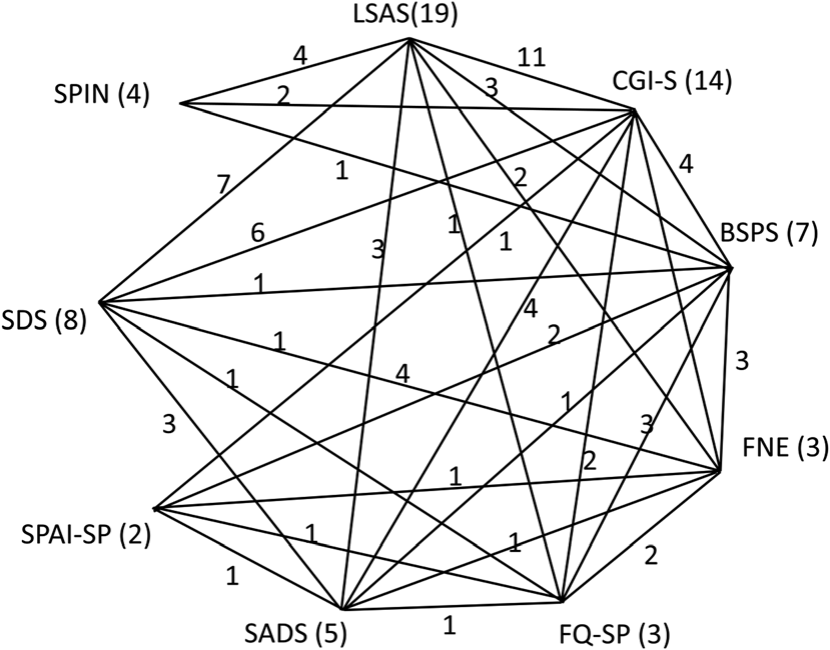
Connected network of test instruments. The numbers indicate the number of trials that the connected tests appeared in together. LSAS, Liebowitz Social Anxiety Scale; CGI-S, Clinicians Global Index—Severity; BPSP, Brief Social Phobia Scale; FNE, Fear of Negative Evaluation; SADS, Social Anxiety and Distress Scale; FQ-SP, Fear Questionnaire—Social Phobia; SPAI-SP, Slocial Phobia Anxiety Index—Social Phobia; SDS, Sheehan Disability Scale; SPIN, Social Phobia Inventory.

It is useful to draw a network diagram showing how many trials report each pair of test outcomes. Figure1 shows that LSAS was the most commonly used test instrument followed by CGI-S, and SPAI-SP the least common. The method proposed requires that each of the tests included are “connected” to one or more other tests by at least one trial.

The data used in the study were the mean treatment differences after 12-week follow-up. Treatment differences and their standard errors were based on mean difference at follow-up if possible or mean difference in change-from-baseline if not. Similarly, the pooled SD at follow-up was used for standardisation if available, otherwise the pooled SD of the change-from-baseline scores (Table 1). Further details of the data extraction and a citation listing of the included studies can be found in the Supporting Information.

**Table 1:** Social anxiety data.

Trials	Sample size	LSAS	CGI-S	BSPS	FNE	FQ-SP	SADS	SPAI-SP	SDS	SPIN

Two-arm trials	Control, Treatment	D (se)	D (se)	D (se)	D (se)	D (se)	D (se)	D (se)	D (se)	D (se)
Kasper (2005)	176, 177	−6.6 (3.21) SD = 30.2								
Asakura (2007)	89, 176	−7.2 (3.07) SD = 16.9							−1.2 (0.72) SD = 5.42	
Davidson (2004)	126, 121	−13.8 (3.05) SD = 28.7	−0.5 (0.14) SD = 1.11						−2.3 (1.00) SD = 7.81	
Westenberg (2004)	148, 146	−8.8 (3.61) SD = 32.6	−0.5 (0.14) SD = 1.21						−2.0 (0.92) SD = 8.47	
Liebowitz (2005a)	138, 133	−8.0 (3.14) SD = 25.8	−0.4 (0.14) SD = 1.16							−3.1 (1.65) SD = 13.62
Rickels (2004)	135, 126	−8.3 (3.79) SD = 30.6	−0.3 (0.16) SD = 1.31							−4.7 (1.97) SD = 15.9
Stein (1998)	92, 90	−21.4 (2.75) SD = 18.6					−4.8 (1.13) SD = 7.63		−2.6 (0.66) SD = 4.47	
Stein (1999)	44, 42	−14.2 (4.56) SD = 22.8		−7.2(2.63) SD = 13.6						−6.7 (3.31) SD = 15.39
Liebowitz (2003)	196, 205	−11.9 (2.79) SD = 28.0	−0.4 (0.12) SD = 1.20	−5.2 (1.45) SD = 14.5						
Allgulander (1999)	48, 44	−24.8 (4.78) SD = 28.7		−11.4 (2.68) SD = 12.5	−6.0 (1.17) SD = 6.89					
Kobak (2002)	30, 30	1.03 (6.62) SD = 25.6		0.4 (3.0) SD = 11.6		−0.54 (2.01) SD = 7.78				
Baldwin (1999)	151, 139	−9.3 (3.96) SD = 33.1	−0.7 (0.30) SD = 1.18				−3.3 (1.41) SD = 8.25			
Pfizer (2007	78, 74	−12.6 (4.24) SD = 16.1	−0.5 (0.18) SD = 1.20						−2.3 (1.13) SD = 7.82	
Lepola (2004)	185, 184	−13.3 (1.86) SD = 17.8	−0.7 (0.11) SD = 1.09				−2.5 (0.74) SD = 7.05		−2.8 (0.35) SD = 3.71	
Van Ameringen (2001)	69, 134		−0.69 (0.13) SD = 0.85	−7.81 (1.85) SD = 12.50	−3.52 (0.85) SD = 5.74	−5.19 (0.91) SD = 6.16	−3.26 (0.88) SD = 5.95	−23.3 (4.19) SD = 28.3		
**Three-arm trials**										

Stein (2005)	134, 131 30	−14.6 (4.41) −14.1 (4.33) SD = 35.5								
Liebowitz (2005b)	144, 133 136	−12.8 (3.62) −17.0 (3.57) SD = 30.6	−0.56 (0.10) −0.73 (0.10) SD = 0.83							
GSK (2006)	130, 133 136	−7.6 (2.47) −6.1 (2.46) SD = 20.0	−0.2 (0.10) −0.2 (0.10) SD = 087							
Allgulander (2004)	132, 129 128	−16.9 (3.36) −16.3 (3.44) SD = 27.4								−8.7 (1.72) −7.7 (1.76) SD = 14.01
Davidson	36, 39 42		−0.60 (0.29) −0.60 (0.29) SD = 1.23	−5.9 (3.09) −5.1 (2.97) SD = 13.1				−25.5 (7.57) −18.7 (6.74) SD = 32.5		
Blomhoff	92, 98 95		−0.59 (0.21) −0.39 (0.21) SD = 1.46	−4.99 (1.37) −2.32 (1.36) SD = 9.38	−2.11(1.37) −0.93(1.23) SD = 8.42	−4.88 (1.24) −4.35 (1.23) SD = 8.52			−4.86 (0.68) −3.28 (0.68) SD = 4.67	
**Four-arm trials**										

Liebowitz (2002)	95, 97 95 97	−9.9 (4.38) −5.3 (4.45) −6.6 (4.41) SD = 30.2	−0.5 (0.18) −0.5 (0.18) −0.5 (0.18) SD = 1.23				−2.2 (1.28) −2.3 (1.30) −2.7 (1.29) SD = 8.80		−1.2 (0.71) −1.3 (0.72) −1.4 (0.71) SD = 4.85	

Each cell gives the mean treatment difference (standard error) relative to placebo for each active arm and pooled standard deviation (SD) at follow-up.

### 2.2. Statistical methods

Bartlett's test of homogeneity of variance (Snedecor and Cochrane, 1980) was applied to the SD data on LSAS in Table 1. Rejection of the null hypothesis of equal variance would indicate that the population SDs in the 19 studies that include LSAS are not the same.

The statistical models used in this study were developed previously to allow for combined estimation of treatment effects on continuous outcomes and “mappings” between treatment effects (Lu *et al*., 2014) in a Bayesian framework. The mappings are simply ratios of the underlying treatment effects. The same model for the treatment effect was used throughout. This was a conventional random-effects model with study effects *δ*_*i*1_ drawn from a normal distribution with mean *μ* and variance *σ*^2^, and *i* denotes study. There is no evidence that these pharmacological treatments differ in efficacy (National Collaborating Centre for Mental Health, 2013), but any variation between treatments would be absorbed into the between-trials variation. In multiarm trials with *M* arms, we draw *M* − 1 treatment effects from the same random-effects distribution.

The index 1 on *δ*_*i*1_ indicates that this is the treatment effect on the LSAS scale; any of the measurement instruments could be selected as outcome 1, but LSAS is chosen as it is the most frequently used. Two models for the relationship between the test instruments were developed. In the first, it is assumed that the treatment effects on test instruments *h* and *k* and *δ*_*ih*_ *and δ*_*ik*_ are in the same fixed ratio in every trial (fixed mapping ratio):


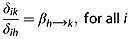
(1)

In a second model (random mapping ratio), this assumption is relaxed, and ratios are allowed to vary around their mean value:


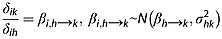
(2)

In the random mapping model, we have proposed a constant between-trials coefficient of variation (CV), *φ*^2^, which is the between-trials SD of mapping coefficients divided by the mean mapping (Lu *et al*., 2014), so that 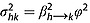. An important feature of both models, which follows from Eqn [1] and [2], is that the ratios must be transitive, so if there are *M* different measurement instruments (in this example, *M* = 9), there are *M*(*M* − 1)/2 ratios to be estimated (in this case, 36), but it is only necessary to estimate *M* − 1 (that is, 8) *basic* mapping parameters (Eddy *et al*., 1992), for example, the ratios of the first with the other *M* − 1, as the remaining 28 are functions of them (Lu *et al*., 2014). Note, however, that the random mapping models are not precisely the same if a different outcome is chosen as the reference. This is taken up further in the Discussion section.

In the Bayesian approach adopted here, we place vague prior distributions on the pooled mean treatment effect, *μ*, the between-trials SD of treatment effects, *σ*^2^, the *M* − 1 mapping coefficients *β*_1 →*h*_, and, in the random mapping ratio model, the between-trials CV *φ*. Technical details of the prior distributions are given in the Supporting Information.

Precisely, the same fixed and random mapping models can be applied to standardised scores:


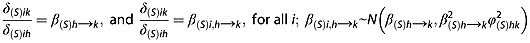


It is important to note that when applied to standardised scores, the pooled estimates represent the treatment effect not simply on a standardised scale, but specifically on the standardised LSAS scale, if LSAS is taken as the reference instrument. A different mean treatment effect on outcome 1 would be obtained if another instrument were chosen as the reference. In this way the model allows for the possibility that different test instruments have different sensitivity to treatment. The mapping coefficients *β*_(*S*)*h* − >*k*_ between standardised treatment effects have a special interpretation which is discussed later. We can test the hypothesis of equal sensitivity by forcing *β*_(*S*)*h* − >*k*_ = 1 for all *h* ≠ *k* and comparing goodness of fit with the model where the ratios are fixed from trial to trial, but not necessarily fixed at one. Further, if standardisation did not introduce additional heterogeneity or “severe distortions” (Rothman *et al*., 2008) we would expect the standardised and non-standardised models to have an identical fit, and more importantly, identical posterior precision of the pooled estimate.

The likelihood for the data is complex. Firstly, the correlations between the original measurements have to be taken into account. Because it is rare for trials to report these correlations, we have used the value of 0.65 for all pairs of tests, which is taken from a review of psychometric literature on measures of social anxiety (National Collaborating Centre for Mental Health, 2013). Analyses using values of 0.55 and 0.75 were also examined to assess sensitivity of results to the assumed correlation. The correlations between mean treatment effects can be derived from correlations between original test scores, as described previously (Lu *et al*., 2014; Wei and Higgins 2013a). Also, because the data take the form of differences between drug and placebo, these differences are correlated in multiarm trials; there are also correlations between the treatment effects on different scales in different arms of the same trial. The full details of the correlation structures between mean treatment effects, the multivariate normal likelihood, and the 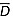 goodness of fit statistic have been described previously in a similar context (Lu *et al*., 2014).

We adopt a Bayesian Markov chain Monte Carlo approach to computation as this affords a flexible approach to fitting models incorporating non-standard functional relationships between variables. The WinBUGS code and its data inputs are set out in the Supporting Information. We compute the mean residual deviance, 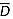, to assess model fit (Spiegelhalter *et al*., 2002). The models appeared to have converged within 5000 to 15 000 iterations, based on the plots of the Brooks and Gelman (1998) statistics available in WinBUGS, with the fixed mapping models converging more rapidly. We also checked that four chains with very different starting values arrived at the same posterior distribution. The results reported in the succeeding texts are based on 20 000 samples each from four chains, having discarded the first 30 000 samples.

## 3. Results

The 19 sample SDs on LSAS (Table 1) range from 16.1 to 35.5, representing a nearly fivefold difference in variance. Bartlett's test gave a *χ*_18_ of 283, allowing us to confidently reject the null hypothesis of equal variance at *p* < 0.001.

Posterior summaries from five models are compared in Table 2. The first three models are based on standardised scores, and the first is estimated under the constraint that all the mapping ratios between standardised treatment effects are 1. The second relaxes this but assumes that mapping ratios are fixed from trial to trial. The third allows the mapping ratios to vary from trial to trial. The pooled mean treatment effect, which is measured on the standardised LSAS scale, and its between-trials SD are very close in these three models. However, the ratios = 1 model has a far worse fit than the fixed mapping model, which is, in turn, a very much worse fit than the random mapping model. In a well-fitting model, 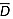 should be approximately equal to the number of observations, which is 88 in this dataset. Under the random mapping model, the degree of between-trials variation in mappings is moderately high with a median between-trials CV of 0.28. This means that the trial-to-trial SD in the mappings is 28% of the mean (95% credible interval (CrI): 19–41%).

**Table 2:** Summary statistics. Treatment effect *μ*: posterior mean and standard deviation in parentheses. All other parameters: posterior medians with 95% credible limits in parentheses.

		Standardisation			No standardisation	
			
		Fixed ratios = 1	Fixed ratios	Random atios	Fixed ratios	Random ratios
Mean treatment effect on LSAS	*μ*	−0.446 (0.0367)	−0.452 (0.0417)	−0.451 (0.0441)	−11.85 (1.016)	−11.71 (1.01)
Between-trial SD	*σ*	0.129 (0.062–0.204)	0.142 (0.082–0.22)	0.156 (0.088–0.24)	3.33 (1.81–5.32)	3.21 (1.62–5.21)
Mappings to LSAS, from CGI-S:	*β*_*LSAS* − >*CGI* − *S*_	—	1.03 (0.91–1.17)	1.05 (0.87–1.29)	0.0443 (0.039–0.050)	0.0445 (0.038–0.052)
BSPS	*β*_*LSAS* − >*BSPS*_	—	0.85 (0.68–1.04)	0.85 (0.61–1.18)	0.373 (0.29–0.46)	0.420 (0.31–0.55)
FNE	*β*_*LSAS* − >*FNE*_	—	0.68 (0.46–0.91)	0.80 (0.50–1.34)	0.186 (0.14–0.35)	0.215 (0.15–0.31)
FQ-SP	*β*_*LSAS* − >*FQ* − *SP*_	—	1.10 (0.85–1.40)	1.14 (0.73–1.91)	0.329 (0.26–0.41)	0.348 (0.25–0.50)
SADS	*β*_*LSAS* − >*SADS*_	—	0.64 (0.50–0.79)	0.70 (0.48–1.03)	0.216 (0.17–0.26)	0.225 (0.17–0.30)
SPAI-SP	*β*_*LSAS* − >*SPAI* − *SP*_	—	1.19 (0.89–1.58)	1.36 (0.82–2.53)	1.53 (1.15–1.99)	1.65 (1.10–2.59)
SDS	*β*_*LSAS* − >*SDS*_		0.99 (0.85–1.15)	0.95 (0.73–1.26)	0.204 (0.18–0.23)	0.207 (0.17–0.27)
SPIN	*β*_*LSAS* − >*SPIN*_		0.92 (0.71–1.14)	0.88 (0.60–1.32)	0.482 (0.37–0.60)	0.478 (0.34–0.66)
Between-trial CV	*φ*	—	—	0.28 (0.19–0.41)		0.18 (0.11–0.29)
Mean residual deviance	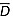	206.2	166.0	86.9	112.2	78.5

The mapping coefficients with standardised data (columns 2 and 3 of Table 2) indicate that, for example, in the random mapping ratio model (column 3), if the standardised treatment effect on LSAS is 1, then the standardised treatment effect on CGI-S is 1.05. This is an average across trials, as the mapping ratios in this model can vary between trials. The interpretation of these coefficients is explored further in the Discussion section.

The results in columns 4 and 5 of Table 2 are from unstandardised data and represent the pooled treatment effect on the LSAS scale and mappings from the LSAS scale to each of the other scales. For example, one-point treatment effect on the LSAS scale would represent a 0.0445-point treatment effect on the CGI-S scale. As with the standardised treatment effects, neither the pooled treatment effect nor its between-trials SD nor the mapping ratios differ greatly between the fixed and random mapping models. However, the identical models applied to non-standardised data fit considerably better than those to standardised data.

Another critical comparison between the standardised and unstandardised analyses is in the relative precision and relative heterogeneity of the estimates. The ratios of the mean treatment effect to their SD, and also the mean treatment effects to the between-trials SDs, are set out in Table 3. The standardisation model with ratios fixed at 1 gives the best results, probably because it has so few parameters to estimate, but this model can be ignored as it fits the data so poorly. Comparing the standardised fixed and random mapping models to their no-standardisation counterparts, it is evident that the latter have better relative precision, by about 7–14% in the relative precision of the estimate and 12–25% in the ratio of treatment effect to between-trials SD. Similarly, the lower between-trials CV in the unstandardised mappings, 18 compared with 28%, attests to the additional noise introduced into the between-trials variation in mapping ratios by standardisation.

**Table 3:** Relative precision of estimates in different models.

Ratio of mean treatment effect to:	Standardisation			No standardisation	
	
	Fixed ratios = 1	Fixed ratios	Random ratios	Fixed ratios	Random ratios
Median standard deviation of mean treatment effect	12.2	10.9	10.2	11.7	11.6
Median between-trials standard deviation	3.5	3.2	2.9	3.6	3.6

Ratios of the posterior mean treatment effect to the posterior standard deviation and ratio of the posterior mean treatment effect to the posterior median between-trials standard deviation.

In Table 4, we illustrate how the mean treatment effect and the between-trials SD of treatment effects can be reported on *any* of the nine test instruments, based on the random mapping model.

**Table 4:** Posterior mean and 95% CrI of the mean treatment effects.

Test instrument	Mean Treatment effect		Between-trials standard deviation	
	
	Mean	95% credible interval	Median	95% credible interval
Liebowitz Social Anxiety Scale	−11.7	−9.8:−13.7	3.20	1.64:5.21
Clinical Global Impression—Severity	−0.523	−0.42:−0.63	0.14	0.07:0.24
Brief Social Phobia Scale	−4.95	−3.55:−6.59	1.34	0.65:2.32
Fear of Negative Evaluation	−2.57	−1.66:−3.72	0.69	0.32:1.24
Fear Questionnaire—Social Phobia	−4.16	−2.84:−5.92	1.11	0.54:2.03
Social Avoidance and Distress Scale	−2.66	−1.87:−3.57	0.72	0.35:1.25
Social Phobia and Anxiety Inventory—Social Phobia	−19.9	−12.7:−30.1	5.26	2.46:10.1
Sheehan Disability Scale	−4.44	−1.88:−3.08	0.66	0.34:1.11
Social Phobia Inventory	−5.66	−3.84:−7.86	1.52	0.72:2.73

Posterior median of the between-trials standard deviation and 95% CrI for each of the nine test instruments, from the random mapping coefficients model.

Sensitivity of results to the correlation between test scores was assessed in the random mapping models, by changing this from the baseline 0.65 to 0.55 and 0.75. This has very little effect on the mean treatment effect, the between-trials variation in treatment effects, or the mapping ratios. Model fit is about three points better with the lower correlation and four to six points worse with the higher correlation. This is to be expected as the higher correlation, in effect, imposes a greater constraint on the data, requiring the treatment effects in different studies to co-vary more closely.

## 4. Discussion

The hypothesis that the nine test instruments used to measure social anxiety in the studies reported here are all equally sensitive to treatment (and therefore have equal reliability and validity), and are thus simple linear transformations of each other, is not plausible. Tests based on different numbers of items must differ in reliability and therefore in sensitivity. Indeed, the theoretical relationship between the number of test items and the reliability of a test has been known for over 100 years (Spearman, 1910). The tests also differ in their content, as shown by factor analyses (Heimberg *et al*., 1999; Baker *et al*., 2002; Saffren *et al*., 1996), which makes exactly equivalent sensitivity a still more remote possibility. In addition, clinician-rated tests must differ from patient-rated tests by introducing inter-observer error. The hypothesis that the mappings between tests are all unity is therefore not credible. Similarly, it seems almost inevitable that division by sample SDs can only introduce further variation. In the illustrative data used here, Bartlett's test on the baseline LSAS variances allows us to reject decisively the null hypothesis that trial populations have the same variance. We can also reject the hypothesis that standardised mappings were all equal to 1. Greenland's comment (1986) that “it is simply misleading to term something a ‘standard unit’ when it is in fact a highly variable quantity” is amply borne out by our results.

The method that we have proposed synthesises the available evidence within and between trials without requiring implausible assumptions. The method takes account of the within-trial correlations, as recommended in the literature on multivariate meta-analysis (Riley, 2009) although these had to be based on external data. This is an important technical improvement on the way in which *within*-trial synthesis of standardised effects is usually carried out. A very common approach is to use a *single* outcome from each trial, based on a preference hierarchy. This would have resulted dropping 58 (66%) of the 88 observed mean differences. Another approach is to take the average of the standardised outcomes on each arm, but this is statistically incorrect unless the correlations are taken into account.

A further problem for standardisation is that some trials report mean treatment effects at follow-up, while others report mean treatment effects on change-from-baseline scores. In the present case, among the 19 trials reporting the LSAS outcome, 7 reported at follow-up and 12 with change scores. With standardisation, one is obliged to either carry out separate analyses or assume that the SDs are not systematically different. In our example, the unweighted arithmetic means of the sample SDs were 27.0 with the follow-up design and 26.6 with the change-score design, so it would seem to be safe to include both designs. However, mapping models based on unstandardised scores can be applied to both types of data without further assumptions.

One of the limitations of the method is that it requires a connected network, such as the one shown in Figure1. It is possible to include trials in which only one outcome is reported: These contribute information to the treatment effect but not on the mappings. However, trials that report *only* outcomes that are not linked to the network cannot be included. A second drawback, which applies to all forms of multivariate meta-analysis, is in the complexity of coding the variance–covariance structures in the likelihood, which is induced by multiarm trials. We are currently addressing this by writing routines to automate this process and thereby supply variance and/or precision matrices for input to different kinds of software. Alternatively, users may enter mean effects on each treatment *arm*, rather than mean treatment *differences*: As the between-arm covariances are all zero, the likelihood is substantially easier to specify.

The random mapping models could be improved on. A drawback of our current formulation is that models with different choice of the reference outcome are not exactly equivalent. A sensitivity analysis in which CGI-S was the reference test instrument, rather than LSAS, had no material effect on the posterior distributions of the mappings, whose medians changed by between 0.5 and 3.4%. These differences represent between 1.2 and 6% of the 95% credible range. However, a model that remains the same under reparameterisation would be preferable.

Two broad classes of alternative approaches can be found in the literature. One is the multivariate normal random effect (MVNRE) meta-analysis model (Riley *et al*., 2007; Jackson *et al*., 2011; Wei and Higgins 2013b; Bujkiewicz *et al*., 2013; Mavridis and Salanti, 2012), which is often suggested for data of the type illustrated in Table 1. MVNRE recognises within-trials and between-trials correlations, and it allows “borrowing strength” across outcomes. However, the impact of data on one outcome on the precision of estimated effects on the other outcomes is usually very limited, and it would be zero if every trial reported on all outcomes. Moreover, MVNRE is not a way of expressing the pooled effects on a common scale, and it does not estimate between-instrument mappings that are required for certain types of cost-effectiveness analysis (National Institute for Health and Care Excellence, 2013).

A second category of methods is perhaps better characterised as heuristics rather than formal statistical methods. Hunter and Schmidt (2004), for example, recognised that variation between trials in SDs created a difficulty for standardisation. They recommended that what they termed “range variation” could be adjusted for by rescaling the sample SDs up or down to a single standard. This is equivalent to using the “standard” SD in all studies, rather than the sample SD. Interestingly, the LSAS is relatively unusual among tests used to measure psychological or psychiatric disorders, in that an SD of 27.5 from 382 individuals from mixed populations (Heimberg *et al*., 1999) is widely considered as a reference standard (the within-studies SD pooled over the 19 studies reporting LSAS here was 27.8). Division of the LSAS mean effects by this reference SD is a form of standardisation that suffers from none of the problems described earlier (Greenland *et al*., 1986; Greenland *et al*., 1991; Rothman *et al*., 2008). By itself, however, this method does not fully address the problem of synthesis of different scales because relatively few test instruments have reference SDs, let alone reference SDs based on the same reference populations.

More recently, a set of proposals has appeared in a number of journals (Thorlund *et al*., 2011; Guyatt *et al*., 2013; Johnston *et al*., 2013), which is in a similar vein to the use of a reference SD. We refer readers to the original papers for a thorough review of the possible approaches. One suggestion is to convert between scales using the ratio of scale ranges as a conversion factor. For example, the LSAS is a 144-point scale, while CGI-S is a seven-point scale. To convert from mean LSAS effects to CGI-S, we could therefore multiply them by 7/144 = 0.0486. This is pleasingly close to the estimated *β*_*LSAS* − >*CGI* − *S*_ mapping ratio of 0.0445 (95% CI: 0.038–0.052) in Table 2. Indeed, the eight conversion factors calculated in this way (CGI-S 0.0486; BSPS 0.50; FNE 0.208; FQ-SP 0.278; SADS 0.194; SPAI-SP 1.33; SDS 0.208; SPIN 0.59) are all within the credible intervals in Table 2. However, the use of range ratios for conversion can be no more than an informal and approximate method because the range of values taken up by a given clinical population may represent different proportions of each instrument's theoretical range and may also vary with population. A further proposal is that mean differences on each scale are converted into “minimal important difference” (MID) units. This suffers from the same problem as reference population SDs, in that few test instruments have recognised MIDs, as well as the fact that MIDs are themselves estimates subject to sampling error and bias in estimation, and they may also depend on the patient's baseline severity (National Collaborating Centre for Mental Health, 2010).

All these proposals, it must be said, have the merit of drawing attention to the importance of understanding relationships between test instruments, the different ways in which results can be reported, and the need for synthesis methods that reflect the functional relationships between results on different test instruments. However, on top of the drawbacks mentioned previously, none of them address the issue of synthesis of evidence *within* trials. More critically, the use of reference SDs, scale ratios, and MID ratios as a means of effecting scale conversions all assumes that the test instruments are equally responsive to the changes brought about by treatments. For example, trials of treatments for anxiety disorders often report results on depression scales, such as the Hamilton (1960) or Beck *et al*. (1961) instruments, because many people with anxiety also have symptoms of depression. Measures of depression are therefore sensitive to changes in anxiety but generally less so than test instruments specifically designed to measure anxiety. The methods proposed here can synthesise information on treatment effects across tests with different sensitivity to treatment, without introducing bias, but this is not the case for the heuristic approaches described previously. This is because the proportion of the total variation that is attributable to patient differences on the construct that is sensitive to treatment depends on both the reproducibility of the test (test–retest reliability and inter-observer error) and its construct validity (Lu *et al*., 2013; Lu *et al*., 2014). As discussed previously, these vary between tests.

The methods proposed here, by contrast, allow for variation between test instruments in sensitivity to treatments. Indeed, the standardised mapping ratios *β*_(*S*)*h* − >*k*_ should be interpreted as direct measures of relative responsiveness of test instruments and specifically their relative responsiveness to treatment. For example, from Table 2, we can estimate, based on the random mapping model, that the SADS instrument is less sensitive to treatment changes than LSAS, by a factor of 0.70 (95% CrI: 0.48–1.03). This means, for example, that a one SD unit treatment effect on LSAS is, on average, equivalent to a 0.70 SD unit effect on SADS. There are, of course, a number of alternative ways of measuring responsiveness of tests, which mainly centre on sensitivity to longitudinal, within-patient, changes (Beaton *et al*., 2001; Terwee *et al*., 2003). However, there are clear advantages in a measure that is based on causal treatment effects from randomised trials, which therefore rule out the influence of confounding factors. There is also merit in a measure that can be estimated meta-analytically, allowing investigators to pool information from as many studies as possible. The use of these methods in the study of relative sensitivity of tests is a project that we are currently pursuing.

Clearly, further experience with these models is required before their properties are sufficiently understood to be recommended for routine use. Among the many questions that need to be answered are how they compare to the standard MVNRE models for multioutcome synthesis and how they might perform if treatment effects were small or non-existent.

## Supporting information

Supporting info item
