# Supplementary material for: Simultaneous synthesis of treatment effects and mapping to a common scale: an alternative to standardisation
Source: Res Synth Methods. 2015 Jan 23;6(1):96–107. doi: 10.1002/jrsm.1130 (PMC4433769; doi:10.1002/jrsm.1130)
Supplement: Supplementary file 1 — Supporting info item [file jrsm0006-0096-sd1.docx]

**SUPPLEMENTARY MATERIAL**

The supplementary material provides citations of trials included, details of the data extraction, an explanation of the prior distributions, and the WinBUGS code, initial values, and datasets

***Trials Included***

1. Allgulander C. Paroxetine in social anxiety disorder: a randomized placebo-controlled study. Acta Psychiatrica Scandinavica. 1999;100:193–98.
2. Allgulander C, Mangano R, Zhang J, Dahl A, Lepola U, Sjodin I, et al. Efficacy of venlafaxine ER in patients with social anxiety disorder: a double-blind, placebo-controlled, parallel-group comparison with paroxetine. Human Psychopharmacology. 2004;19:387–96.
3. Asakura S, Tajima O, Koyama T. Fluvoxamine treatment of generalized social anxiety disorder in Japan: a randomized double-blind, placebo-controlled study. International Journal of Neuropsychopharmacology. 2007;10:263–74.
4. Baldwin DS, Bobes J, Stein DJ, Scharwächter I, Faure M. Paroxetine in social phobia/ social anxiety disorder. Randomised, double-blind, placebo-controlled study. Paroxetine Study Group. The British Journal of Psychiatry. 1999;175:120–26.
5. Blomhoff S, Haug TT, Hellstrom K, Holme I, Humble M, Madsbu HP, et al. Randomised controlled general practice trial of sertraline, exposure therapy and combined treatment in generalised social phobia. British Journal of Psychiatry. 2001;179:23–30.
6. Davidson J, Yaryura-Tobias J, DuPont R, Stallings L, Barbato L, van der Hoop R, et al. Fluvoxamine-controlled release formulation for the treatment of generalized social anxiety disorder. Journal of Clinical Psychopharmacology. 2004a;24:118–25.
7. Davidson JR, Foa EB, Huppert JD, Keefe FJ, Franklin ME, Compton JS, et al. Fluoxetine, comprehensive cognitive behavioral therapy, and placebo in generalized social phobia. Archives of General Psychiatry. 2004b;61:1005–13.
8. GlaxoSmithKline. A randomized, double-blind, fixed dose comparison of 20, 40, and 60 mg daily of paroxetine and placebo in the treatment of generalized social phobia. GSK Clinical Study Register; 1997.
9. Kasper S, Stein DJ, Loft H, Nil R. Escitalopram in the treatment of social anxiety disorder: randomised, placebo-controlled, flexible-dosage study. British Journal of Psychiatry. 2005;186:222–26.
10. Kobak KA, Greist JH, Jefferson JW, Katzelnick DJ. Fluoxetine in social phobia: a double-blind, placebo-controlled pilot study. Journal of Clinical Psychopharmacology. 2002;22:257–62.
11. Lepola U, Bergtholdt B, St Lambert J, Davy KL, Ruggiero L. Controlled-release paroxetine in the treatment of patients with social anxiety disorder. Journal of Clinical Psychiatry. 2004;65:222–29.
12. Liebowitz MR, Stein MB, Tancer M, Carpenter D, Oakes R, Pitts CD. A randomized, double-blind, fixed-dose comparison of paroxetine and placebo in the treatment of generalized social anxiety disorder. Journal of Clinical Psychiatry. 2002b;63:66–74.
13. Liebowitz MR, DeMartinis NA, Weihs K, Londborg PD, Smith WT, Chung H, et al. Efficacy of sertraline in severe generalized social anxiety disorder: results of a double-blind, placebo-controlled study. Journal of Clinical Psychiatry. 2003;64:785–92.
14. Liebowitz MR, Gelenberg AJ, Munjack D. Venlafaxine extended release vs placebo and paroxetine in social anxiety disorder. Archives of General Psychiatry. 2005a;62:190–98.
15. Liebowitz MR, Mangano RM, Bradwejn J, Asnis G. A randomized controlled trial of venlafaxine extended release in generalized social anxiety disorder. Journal of Clinical Psychiatry. 2005b;66:238–47.
16. Pfizer. A 10-week, randomized, double-blind, placebo-controlled study of paroxetine and pregabalin in patients with social phobia (1008-081 and 1008-153). Web Synopsis Protocol 1008-081/153 - 21 June 2007 - Final.
17. Rickels K, Mangano R, Khan A. A double-blind, placebo-controlled study of a flexible dose of venlafaxine ER in adult outpatients with generalized social anxiety disorder. Journal of Clinical Psychopharmacology. 2004;24:488–96.
18. Stein MB, Liebowitz MR, Lydiard RB, Pitts CD, Bushnell W, Gergel I. Paroxetine treatment of generalized social phobia (social anxiety disorder): a randomized controlled trial. Journal of the American Medical Association. 1998b;280:708–13.
19. Stein MB, Fyer AJ, Davidson JR, Pollack MH, Wiita B. Fluvoxamine treatment of social phobia (social anxiety disorder): a double-blind, placebo-controlled study. American Journal of Psychiatry. 1999a;156:756–60.
20. Stein MB, Pollack MH, Bystritsky A, Kelsey JE, Mangano RM. Efficacy of low and higher dose extended-release venlafaxine in generalized social anxiety disorder: a 6-month randomized controlled trial. Psychopharmacology. 2005; 177:280–88.
21. Van Ameringen MA, Lane RM, Walker JR, Bowen RC, Chokka PR, Goldner EM, et al. Sertraline treatment of generalized social phobia: a 20–week, double-blind, placebo-controlled study. American Journal of Psychiatry. 2001;158:275–81.
22. Westenberg HG, Stein DJ, Yang H, Li D, Barbato LM. A double-blind placebo-controlled study of controlled release fluvoxamine for the treatment of generalized social anxiety disorder. Journal of Clinical Psychopharmacology. 2004;24:49–55.

***Data extraction***

In studies where neither standard deviations nor standard errors were available on outcomes, a standard error was inferred from 95% confidence intervals on mean treatment differences, or from an exact *p*-value, via a *t*-statistic, on the basis that the SD was equal in both arms.

In one study, only the range (maximum minus minimum) was recorded. The expected SD given the range and the sample size was estimated by simulation. The SD of the CGI-S was calculated from the distribution of raw scores, if that was the only information available.

Other special calculations that were made are as follows:

*Asakura (2007).* In the text, the mean treatment effect is positive, making placebo more effective than treatment. Both the wording in the discussion and abstract, and Figure 3, indicate the opposite. We have therefore made the effect negative in the analysis.

*Van Ameringen (2001).* The standard deviation of changes at endpoint scores given in Table 2 appeared implausible as they were only 25% or less of the reported SD of baseline scores. We therefore used the 95%CIs for the mean treatment effect, which gave SDs much closer to those reported at baseline.

*Blomhoff (2001).* The mean change (follow-up minus baseline) on BSPS in the sertraline arm reported in Table 3 is not consistent with the mean change on placebo and the mean treatment effect reported in the same table. We have used the “mean change v. placebo”, as this is consistent with the reported confidence limits.

***Prior Distributions***

Priors for the treatment effect mean and between-trials variation are $\mu\sim N(0,1000^{2})$ and$\sigma\sim U(0,10)$. The between-trial variance in mappings was given an vague inverse Gamma prior , but truncated to have a maximum value of 1. This blocks CVs above 1. It can be confirmed from Figure S1 that this does not impose unreasonable limits on the posterior. With regard to priors for mapping coefficients, the present dataset is particularly sparse, with 4 mappings informed from only 4 or fewer trials. We found that carefully constructed priors were required to prevent slow convergence, convergence to different posteriors, and posteriors having very long flat tails to the right. We therefore centred each prior on the ratios of the ranges, that is the maximum score minus the minimum score, of the test instruments. For example, the ranges are: LSAS 144, CGI-S 7, BSPS 72, FNE 30, SADS 28, FQ-SP 40, SPAI-SP 192, SDS 30, SPIN 85. This gives ratios as follows: CGI-S : LSAS 0.048, BSPS : LSAS 0.50, FNE: LSAS 0.21, SADS : LSAS 0.19, FQ-SP : LSAS 0.28, SPAI-SP : LSAS 1.33, SDS : LSAS 0.21, SPIN : LSAS 0.59. If we label these “scale ratios” as *S_k_* then the priors were:

$$Abs(\beta_{k->LSAS})\sim N(S_{k},(3.5S_{k})^{2})\text{ }I(0,5S_{k})$$

With standardized models *S_k_* is set to 1.

Note that the prior standard deviations are 3.5 times the scale ratios, and that an absolute upper limit of 5 times the scale ratios is also imposed. Note, also, that the sign of the mapping is assumed to be “known” in all cases, so that the absolute value of the mapping is limited to zero. In this dataset, for example, a higher score was associated with a worsening of the condition in every test, so that mapping ratios and correlations are all forced to be positive. In the WinBUGS code this is achieved by drawing both mean and trial -specific mappings from normal distributions, but then using variables with fixed signs to define the likelihoods, constructed for example as follows: sbeta.m[k]<-sign[k]*abs(beta.m[k]) . This approach will allow synthesis of tests that go in opposite directions (Lu et al., 2014).

The posterior densities of the mean mapping rations in the Random Mapping model without standardisation (Figure S1) show that the above prior is not causing undue truncation. Further, sensitivity analyses show that widening the prior variance to $(4S_{k})^{2}$ and extending the upper bound to$6S_{k}$has no material effect on posteriors.

Figure S1. Posterior distributions from the Random mapping model with no standardization. (a) Mean treatment effect (mu), (b,c,d,e,f,g,h,i) mapping ratios from LSAS to: CGI-S, BSPS, FNE, FQ-SP, SADS, SPAI-SP, SDS, SPIN; (j) between-trials coefficient of variation in mappings; (k) between-trials standard deviation

(a)

(d)

(g)

(b)

(e)

(c)

(h)

(j)

(f)

(i)

(k)

***WinBUGS Implementation***

Below we set out the WinBUGS code, initial values and data for the Random Effects model with standardization. We then show how the code and data are modified for analyses on unstandardized variables, and how Fixed Effect models are coded

**Social Anxiety RE mapping model with standardization**

model {

**# 15 2-arm trials**

for(i in 1:15) { delta[i]~dnorm(mu, tau) } # random trt effect in trial i on outcome 1 (LSAS)

#corr between measures

rho<-0.65

#rho<-0.55 # values used in sensitivity analyses

#rho<-0.75

beta.m[1]<-1 # MAPPINGS

sbeta.m[1]<-1

for(k in 2:9) { P[k] <- pow(3.5*S[k],-2)

L[k] <- 5*S[k]

beta.m[k] ~ dnorm(S[k],P[k]) I(,L[k])

sbeta.m[k]<-sign[k]*abs(beta.m[k]) # signed mean mappings

tau.m[k]<-1/pow(sig.m[k],2) # precision of mappings

sig.m[k]<-abs(beta.m[k])*sig.m1 } # sd of mappings

for (k in 1:9) { sign[k]<-1 } # all mappings are positive

for(i in 1:22) {

beta[i,1]<-1

sbeta[i,1]<-sign[1]

for(k in 2:9) { beta[i,k] ~ dnorm(beta.m[k], tau.m[k]) # trial specific mappings

sbeta[i,k]<-sign[k]*abs(beta[i,k]) } # signed trial specific mappings

}

tau.m1~dgamma(.005,.005) I(1,)  # vague prior for trial mappings

sig.m1 <- pow(tau.m1,-0.5)

mu~dnorm(0,.001) # vague prior for mean treatment effect

tau<-1/pow(sig,2)

sig~dunif(0,10) # vague prior for between-trial variation in treatment effect

# generate mean treatment effects on every instrument

# ... and between-trial sds on every instrument

for (k in 2:9) {mm[k] <- mu * sbeta.m[k]

sd[k] <- sig * abs(beta.m[k]) }

**# LIKELIHOODS for Cohen's d,** indexation *i* for trial

# 2-ARM TRIALS

# i=1:1 one 1-dim

for(i in 1:1){

ds[i,1] <- d[i,1]/SD[i,1]

vCs[i,1] <- vC[i,1]/pow(SD[i,1],2)

vTs[i,1] <- vT[i,1]/pow(SD[i,1],2)

ds[i,1]~dnorm(mu1[i], prec1[i])

prec1[i]<-1/w1[i]

mu1[i]<-delta[i]*sbeta[i,t[i,1]]

w1[i]<-vCs[i,1]/nC[i] + vTs[i,1]/nT[i]

res[i,1]<-ds[i,1]-mu1[i]

dev[i]<-pow(res[i,1], 2) * prec1[i]

}

# i=1:2 one (2-dim)

for(i in 2:2){

for(j in 1:2){ds[i,j]<-d[i,j]/SD[i,j]

vCs[i,j]<-vC[i,j]/pow(SD[i,j],2)

vTs[i,j]<-vT[i,j]/pow(SD[i,j],2) }

ds[i,1:2]~dmnorm(mu2[i,1:2], prec2[i,1:2,1:2]) # MVN likelihood

prec2[i,1:2,1:2]<-inverse(w2[i,1:2,1:2])

for(j in 1:2){

mu2[i,j]<- delta[i ]* sbeta[i,t[i,j]] # mean

w2[i,j,j]<-(vCs[i,j]/nC[i]) + (vTs[i,j]/nT[i])} # variance of diagonal terms

for(k in 1:1){

for(h in (k+1):2) {w2[i,k, h]<- (sqrt(vCs[i,k]*vCs[i,h])/nC[i] + # covariance

sqrt(vTs[i,h]*vTs[i,k])/nT[i] ) * rho

w2[i,h, k]<-w2[i,k, h] }}

for(k in 1:2){ res[i, k]<-ds[i,k]-mu2[i,k] } # residuals

for(k in 1:2){ m[i,k]<-inprod(prec2[i,k,1:2], res[i,1:2]) }

dev[i]<-inprod(m[i,1:2],res[i,1:2]) # deviance for MVN likelihood

}

# i = 3:13 eleven (3-dim)

for(i in 3:13) {

for(j in 1:3){ds[i,j]<-d[i,j]/SD[i,j]

vCs[i,j]<-vC[i,j]/pow(SD[i,j],2)

vTs[i,j]<-vT[i,j]/pow(SD[i,j],2) }

ds[i,1:3]~dmnorm(mu3[i,1:3], prec3[i,1:3,1:3])

prec3[i,1:3,1:3]<-inverse(w3[i,1:3,1:3])

for(j in 1:3){

mu3[i,j]<- delta[i ]* sbeta[i,t[i,j]]

w3[i,j,j]<-(vCs[i,j]/nC[i]) + (vTs[i,j]/nT[i])}

for(k in 1:2){

for(h in (k+1):3){w3[i,k, h]<- (sqrt(vCs[i,k]*vCs[i,h])/nC[i] +

sqrt(vTs[i,h]*vTs[i,k])/nT[i]) * rho

w3[i,h, k]<-w3[i,k, h] }}

for(k in 1:3){ res[i, k]<-ds[i,k]-mu3[i,k] }

for(k in 1:3){ m[i,k]<-inprod(prec3[i,k,1:3], res[i,1:3]) }

dev[i]<-inprod(m[i,1:3],res[i,1:3])

}

# i= 14:14 0ne (4-dim)

for(i in 14:14){

for(j in 1:4){ds[i,j]<-d[i,j]/SD[i,j]

vCs[i,j]<-vC[i,j]/pow(SD[i,j],2)

vTs[i,j]<-vT[i,j]/pow(SD[i,j],2) }

ds[i,1:4]~dmnorm(mu4[i,1:4], prec4[i,1:4,1:4])

prec4[i,1:4,1:4]<-inverse(w4[i,1:4,1:4])

for(j in 1:4){

mu4[i,j]<- delta[i ]* sbeta[i,t[i,j]]

w4[i,j,j]<-(vCs[i,j]/nC[i]) + (vTs[i,j]/nT[i])}

for(k in 1:3){

for(h in (k+1):4){w4[i,k,h] <- (sqrt(vCs[i,k]*vCs[i,h])/nC[i] +

sqrt(vTs[i,h]*vTs[i,k])/nT[i]) * rho

w4[i,h,k] <- w4[i,k,h] }}

for(k in 1:4) { res[i,k] <- ds[i,k]-mu4[i,k] }

for(k in 1:4) { m[i,k] <- inprod(prec4[i,k,1:4], res[i,1:4]) }

dev[i]<-inprod(m[i,1:4],res[i,1:4])

}

# i= 15:15 0ne (6-dim)

for (i in 15:15) {

for(j in 1:6){ds[i,j]<-d[i,j]/SD[i,j]

vCs[i,j]<-vC[i,j]/pow(SD[i,j],2)

vTs[i,j]<-vT[i,j]/pow(SD[i,j],2) }

ds[i,1:6] ~ dmnorm(mu6[i,1:6], prec6[i,1:6,1:6])

prec6[i, 1:6,1:6]<-inverse(w6[i,1:6,1:6])

for(j in 1:6){

mu6[i,j] <- delta[i ]* sbeta[i,t[i,j]]

w6[i, j,j] <- (vCs[i,j]/nC[i]) + (vTs[i,j]/nT[i])}

for(k in 1:5){

for(h in (k+1):6) {w6[i,k,h] <- (sqrt(vCs[i,k]*vCs[i,h])/nC[i] +

sqrt(vTs[i,h]*vTs[i,k])/nT[i] ) * rho

w6[i,h,k] <- w6[i,k,h] }}

for(k in 1:6) { res[i,k] <- ds[i,k]-mu6[i,k] }

for(k in 1:6) { m[i,k] <- inprod(prec6[i,k,1:6], res[i,1:6]) }

dev[i]<-inprod(m[i,1:6],res[i,1:6])

}

**# 3-ARM TRIALS**

**for(i in 1:6)**{ for(j in 1:2) {a[i,j] ~ dnorm(0,tau) } # MVN between-arm model

ddelta[i,1]<- mu + 0.5*(sqrt(3)*a[i,1]+a[i,2])

ddelta[i,2]<- mu + 0.5*(sqrt(3)*a[i,1] -a[i,2])

}

# i=1:1 one (1-dim), trial 16

for(i in 1:1){

d2s1[i,1] <- d2[i,1]/SD[i+15,1]

d2s1[i,2] <- d2[i,6] / SD[i+15,1]

vC2s1[i,1] <- vC2[i,1] / pow(SD[i+15,1],2)

vT2s1[i,1] <- vT2[i,1] / pow(SD[i+15,1],2)

vT2s1[i,6] <- vT2[i,6] / pow(SD[i+15,1],2)

d22[i,1]<-d2s1[i,1]

d22[i,2]<-d2s1[i,2]

d22[i,1:2]~dmnorm(mu22[i,1:2],prec22[i,1:2,1:2])

prec22[i,1:2,1:2]<-inverse(w22[i,1:2,1:2])

w22[i,2,1]<-w22[i,1,2] #non-diagonal

w22[i,1,2]<-vC2s1[i,1]/nC2[i]

w22[i,1,1]<-vC2s1[i,1]/nC2[i] + vT2s1[i,1]/nT2[i,1] #diagonal

w22[i,2,2]<-vC2s1[i,1]/nC2[i] + vT2s1[i,6]/nT2[i,2] #diagonal

for(k in 1:2){

mu22[i,k]<-ddelta[i,k]*sbeta[15+i, t2[i,1]]

res[15+i,k]<-d22[i,k]-mu22[i,k]

m[15+i,k]<-inprod(prec22[i,k,1:2],res[15+i,1:2])

}

dev[15+i]<-inprod(m[15+i,1:2],res[15+i,1:2])

}

# i=2:4 3-arm, 2-outcome, trials 17,18,19

for(i in 1:3){

for(j in 1:2){ d2s2[i,j]<-d2[i+1,j] / SD[i+16,j]

d2s2[i,j+2]<-d2[i+1,j+5] / SD[i+16,j]

vC2s2[i,j]<-vC2[i+1,j] / pow(SD[i+16,j],2)

vT2s2[i,j]<-vT2[i+1,j] / pow(SD[i+16,j],2)

vT2s2[i,j+2]<-vT2[i+1,j+5] / pow(SD[i+16,j],2) }

d24[i,1]<-d2s2[i,1]

d24[i,2]<-d2s2[i,2]

d24[i,3]<-d2s2[i,3]

d24[i,4]<-d2s2[i,4]

d24[i,1:4]~dmnorm(mu24[i,1:4],prec24[i,1:4,1:4])

prec24[i, 1:4,1:4]<-inverse(w24[i,1:4,1:4])

for(k in 1:3){for(j in (k+1):4){

w24[i,j,k]<-w24[i,k,j] }

}

for(k in 1:2){

mu24[i,k]<-ddelta[i+1,1]*sbeta[16+i, t2[i+1,k]]

mu24[i,2+k]<-ddelta[i+1,2]*sbeta[16+i,t2[i+1,k]]

w24[i,k,k]<-vC2s2[i,k]/nC2[i+1] + vT2s2[i,k]/nT2[i+1,1]

w24[i,k+2,k+2]<-vC2s2[i,k]/nC2[i+1] + vT2s2[i,k+2]/nT2[i+1,2] #diagonal

res[16+i,k]<-d24[i,k]-mu24[i,k]

res[16+i,k+2]<-d24[i,k+2]-mu24[i, k+2]

}

for(k in 1:4){ m[16+i, k]<-inprod(prec24[i,k,1:4],res[16+i,1:4]) }

dev[16+i]<-inprod(m[16+i,1:4],res[16+i,1:4])

w24[i,1,3]<-vC2s2[i,1]/nC2[i+1]

w24[i,2,4]<-vC2s2[i,2]/nC2[i+1]

w24[i,1,2]<-rho*(sqrt(vC2s2[i,1]*vC2s2[i,2])/nC2[i+1]+ sqrt(vT2s2[i,1]*vT2s2[i,2])/nT2[i+1,1])

w24[i,3,4]<-rho*(sqrt(vC2s2[i,2]*vC2s2[i,1])/nC2[i+1]+ sqrt(vT2s2[i,3]*vT2s2[i,4])/nT2[i+1,2])

w24[i,1,4]<-rho*sqrt(vC2s2[i,1]*vC2s2[i,2]) / nC2[i+1]

w24[i,2,3]<-rho*sqrt(vC2s2[i,1]*vC2s2[i,2]) / nC2[i+1]

}

# i in 5:5 one 3-arm, 3-outcome, Trial 20

for(i in 1:1){

for(j in 1:3){ d2s3[i,j]<-d2[i+4,j]/SD[i+**19**,j]

d2s3[i,j+3]<-d2[i+4,j+5]/SD[i+19,j]

vC2s3[i,j]<-vC2[i+4,j]/pow(SD[i+19,j],2)

vT2s3[i,j]<-vT2[i+4,j]/pow(SD[i+19,j],2)

vT2s3[i,j+3]<-vT2[i+4,j+5]/pow(SD[i+19,j],2) }

for(k in 1:3) { d26[i,k]<-d2s3[i,k] } #6-dim

for (k in 1:3) {d26[i,k+3] <- d2s3[i,k+3] }

d26[i,1:6]~dmnorm(mu26[i,1:6],prec26[i,1:6,1:6])

prec26[i,1:6,1:6]<-inverse(w26[i,1:6,1:6])

for(k in 1:5){for(j in (k+1):6){ w26[i,j,k]<-w26[i,k,j] }}

for(k in 1:3){

mu26[i,k]<-ddelta[i+4,1]*sbeta[19+i, t2[i+4,k]]

mu26[i,3+k]<-ddelta[i+4,2]*sbeta[19+i,t2[i+4,k]]

w26[i,k,k]<-vC2s3[i,k]/nC2[i+4] + vT2s3[i,k]/nT2[i+4,1]

w26[i,k+3,k+3]<-vC2s3[i,k]/nC2[i+4] + vT2s3[i,k+3]/nT2[i+4,2] #diagonal

res[19+i,k]<-d26[i,k]-mu26[i,k]

res[19+i, k+3]<-d26[i,k+3]-mu26[i,k+3]

}

for(k in 1:6){ m[19+i, k]<-inprod(prec26[i,k,1:6],res[19+i,1:6]) }

dev[19+i]<-inprod(m[19+i,1:6],res[19+i,1:6])

for(k in 1:3){ w26[i,k,k+3]<-vC2s3[i,k]/nC2[i+4] }

w26[i,1,5]<-rho*sqrt(vC2s3[i,1]*vC2s3[i,2]) /nC2[i+4]

w26[i,1,6]<-rho*sqrt(vC2s3[i,1]*vC2s3[i,3]) /nC2[i+4]

w26[i,2,4]<-rho*sqrt(vC2s3[i,2]*vC2s3[i,1]) /nC2[i+4]

w26[i,2,6]<-rho*sqrt(vC2s3[i,2]*vC2s3[i,3]) /nC2[i+4]

w26[i,3,4]<-rho*sqrt(vC2s3[i,3]*vC2s3[i,1]) /nC2[i+4]

w26[i,3,5]<-rho*sqrt(vC2s3[i,3]*vC2s3[i,2]) /nC2[i+4]

for(k in 1:2){for(h in (k+1):3){

w26[i,k,h]<-rho*(sqrt(vC2s3[i,k]*vC2s3[i,h])/nC2[i+4]+sqrt(vT2s3[i,k]*vT2s3[i,h])/nT2[i+4,1])

w26[i,k+3,h+3]<-rho*(sqrt(vC2s3[i,k]*vC2s3[i,h])/nC2[i+4]

+ sqrt(vT2s3[i,k+3]*vT2s3[i,h+3])/nT2[i+4,2] ) }}

}

# i=6:6 One 3-arm 5-outcome Trial 21

for (i in 1:1) {

for(j in 1:5){ d2s4[i,j]<-d2[i+5,j]/SD[20+i,j]

d2s4[i,j+5]<-d2[i+5,j+5]/SD[20+i,j]

vC2s4[i,j]<-vC2[i+5,j]/pow(SD[20+i,j],2)

vT2s4[i,j]<-vT2[i+5,j]/pow(SD[20+i,j],2)

vT2s4[i,j+5]<-vT2[i+5,j+5]/pow(SD[20+i,j],2) }

for(k in 1:10){ d210[i,k]<-d2s4[i,k] } #10-dim

d210[i,1:10] ~ dmnorm(mu210[i,1:10], prec210[i,1:10,1:10])

prec210[i,1:10,1:10]<-inverse(w210[i,1:10,1:10])

for (k in 1:9) { for (j in (k+1):10) {w210[i,j,k]<-w210[i,k,j] }}

for (k in 1:5) {

mu210[i,k]<-ddelta[i+5,1]*sbeta[20+i, t2[i+5,k]]

mu210[i,5+k]<-ddelta[i+5,2]*sbeta[20+i, t2[i+5,k]]

w210[i,k,k]<-vC2s4[i,k]/nC2[i+5] + vT2s4[i,k]/nT2[i+5,1] # diagonal

w210[i,k+5,k+5]<-vC2s4[i,k]/nC2[i+5] + vT2s4[i,k+5]/nT2[i+5,2] # diagonal

res[20+i,k]<-d210[i,k]-mu210[i,k]

res[20+i,k+5]<-d210[i,k+5]-mu210[i,k+5]

}

for (k in 1:10) { m[20+i,k]<-inprod(prec210[i,k,1:10], res[20+i,1:10])}

dev[20+i]<-inprod(m[20+i,1:10],res[20+i,1:10])

# same outcome, different arms

for (k in 1:5) {w210[i, k,k+5]<-vC2s4[i,k]/nC2[i+5] }

# same arm, different outcomes

for (k in 1:4) {

for (h in (k+1):5) { w210[i,k,h]<-rho*(sqrt(vC2s4[i,k]*vC2s4[i,h])/nC2[i+5]

+sqrt(vT2s4[i,k]*vT2s4[i,h])/nT2[i+5,1])

w210[i,k+5,h+5]<-rho*(sqrt(vC2s4[i,k]*vC2s4[i,h])/nC2[i+5]

+sqrt(vT2s4[i,k+5]*vT2s4[i,h+5])/nT2[i+5,2])

}

}

# different arms, different outcomes

w210[i, 1,7]<-rho*sqrt(vC2s4[i,2]*vC2s4[i,1])/nC2[i+5];

w210[i, 1,8]<-rho*sqrt(vC2s4[i,3]*vC2s4[i,1])/nC2[i+5];

w210[i, 1,9]<-rho*sqrt(vC2s4[i,4]*vC2s4[i,1])/nC2[i+5];

w210[i, 1,10]<-rho*sqrt(vC2s4[i,5]*vC2s4[i,1])/nC2[i+5];

w210[i, 2,6]<-rho*sqrt(vC2s4[i,1]*vC2s4[i,2])/nC2[i+5];

w210[i, 2,8]<-rho*sqrt(vC2s4[i,3]*vC2s4[i,2])/nC2[i+5];

w210[i, 2,9]<-rho*sqrt(vC2s4[i,4]*vC2s4[i,2])/nC2[i+5];

w210[i, 2,10]<-rho*sqrt(vC2s4[i,5]*vC2s4[i,2])/nC2[i+5];

w210[i, 3,6]<-rho*sqrt(vC2s4[i,1]*vC2s4[i,3])/nC2[i+5];

w210[i, 3,7]<-rho*sqrt(vC2s4[i,2]*vC2s4[i,3])/nC2[i+5];

w210[i, 3,9]<-rho*sqrt(vC2s4[i,4]*vC2s4[i,3])/nC2[i+5];

w210[i, 3,10]<-rho*sqrt(vC2s4[i,5]*vC2s4[i,3])/nC2[i+5];

w210[i, 4,6]<-rho*sqrt(vC2s4[i,1]*vC2s4[i,4])/nC2[i+5];

w210[i, 4,7]<-rho*sqrt(vC2s4[i,2]*vC2s4[i,4])/nC2[i+5];

w210[i, 4,8]<-rho*sqrt(vC2s4[i,3]*vC2s4[i,4])/nC2[i+5];

w210[i, 4,10]<-rho*sqrt(vC2s4[i,5]*vC2s4[i,4])/nC2[i+5];

w210[i, 5,6]<-rho*sqrt(vC2s4[i,1]*vC2s4[i,5])/nC2[i+5];

w210[i, 5,7]<-rho*sqrt(vC2s4[i,2]*vC2s4[i,5])/nC2[i+5];

w210[i, 5,8]<-rho*sqrt(vC2s4[i,3]*vC2s4[i,5])/nC2[i+5];

w210[i, 5,9]<-rho*sqrt(vC2s4[i,4]*vC2s4[i,5])/nC2[i+5];

}

# One 4-arm 4-outcome Trial 22, 12-D

for (j in 1:3) { aa[j] ~ dnorm(0,tau) } # four-arm between trial model

dddelta[1]<- mu + sqrt(2/3)*aa[1]+sqrt(1/3)*aa[3]

dddelta[2]<- mu + sqrt(2/3)*aa[1]+0.5*aa[2]-sqrt(1/12)*aa[3]

dddelta[3]<- mu + sqrt(2/3)*aa[1]-0.5*aa[2]-sqrt(1/12)*aa[3]

for(j in 1:4){ d3s[j] <- d3[j]/SD[22,j]

d3s[j+4] <- d3[j+4]/SD[22,j]

d3s[j+8] <- d3[j+8]/SD[22,j]

vC3s[j] <- vC3[j]/pow(SD[22,j],2)

vT3s[j] <- vT3[j]/pow(SD[22,j],2)

vT3s[j+4] <- vT3[j+4]/pow(SD[22,j],2)

vT3s[j+8] <- vT3[j+8]/pow(SD[22,j],2) }

for (j in 1:12) { d312[j] <- d3s[j] }

d312[1:12] ~ dmnorm(mu312[1:12], prec312[1:12,1:12])

prec312[1:12,1:12]<-inverse(w312[1:12,1:12])

for (k in 1:11) { for (j in (k+1):12) {w312[j,k]<-w312[k,j] }}

for (k in 1:4) {

mu312[k] <- dddelta[1]*sbeta[22,t3[k]]

mu312[k+4] <- dddelta[2]*sbeta[22,t3[k]]

mu312[k+8] <- dddelta[3]*sbeta[22,t3[k]]

w312[k,k] <- vC3s[k] / nC3 + vT3s[k] / nT3[1] # diagonal

w312[k+4,k+4] <- vC3s[k] / nC3 + vT3s[k+4] / nT3[2] # diagonal

w312[k+8,k+8] <- vC3s[k] / nC3 + vT3s[k+8] / nT3[3] # diagonal

}

for (k in 1:12) {

res[22,k]<-d312[k] - mu312[k]

m[22,k]<-inprod(prec312[k,1:12],res[22,1:12])

}

dev[22]<-inprod(m[22,1:12], res[22, 1:12])

# same outcome, different arms

for (k in 1:4) { w312[k,k+4] <- vC3s[k]/nC3}

for (k in 5:8) { w312[k,k+4] <- vC3s[k-4]/nC3}

for (k in 1:4) { w312[k,k+8] <- vC3s[k]/nC3}

# same arm, different outcomes

for (k in 1:3) {

for (h in (k+1):4) {

w312[k,h] <- rho * (sqrt(vC3s[k]*vC3s[h])/nC3 + sqrt(vT3s[k]*vT3s[h]) / nT3[1])

w312[k+4,h+4] <- rho * (sqrt(vC3s[k] * vC3s[h]) / nC3

+ sqrt(vT3s[k+4] * vT3s[h+4]) / nT3[2])

w312[k+8,h+8]<- rho * (sqrt(vC3s[k] * vC3s[h]) / nC3

+ sqrt(vT3s[k] * vT3s[h+8]) / nT3[3])

}

}

# different arms, different outcomes

w312[1,6] <- rho *sqrt(vC3s[2] *vC3s[1])/nC3

w312[1,7] <- rho *sqrt(vC3s[3] *vC3s[1])/nC3

w312[1,8] <- rho * sqrt(vC3s[4] *vC3s[1])/nC3

w312[2,5] <-rho *sqrt(vC3s[1] * vC3s[2])/nC3

w312[2,7] <-rho *sqrt(vC3s[3] *vC3s[2])/nC3

w312[2,8] <-rho *sqrt(vC3s[4]* vC3s[2])/nC3

w312[3,5] <-rho * sqrt(vC3s[1] *vC3s[3])/nC3

w312[3,6] <-rho *sqrt(vC3s[2] *vC3s[3])/nC3

w312[3,8] <-rho *sqrt(vC3s[4] *vC3s[3])/nC3

w312[4,5] <- rho *sqrt(vC3s[1] *vC3s[4])/nC3

w312[4,6] <-rho *sqrt(vC3s[2] *vC3s[4])/nC3

w312[4,7] <-rho *sqrt(vC3s[3] *vC3s[4])/nC3

## end of 1st arm and 2-arm

w312[1,10] <-rho *sqrt(vC3s[2] *vC3s[1])/nC3

w312[1,11] <-rho *sqrt(vC3s[3] *vC3s[1])/nC3

w312[1,12] <-rho *sqrt(vC3s[4] *vC3s[1])/nC3

w312[2,9] <-rho *sqrt(vC3s[1] *vC3s[2])/nC3

w312[2,11] <-rho *sqrt(vC3s[3] *vC3s[2])/nC3

w312[2,12] <-rho *sqrt(vC3s[4] *vC3s[2])/nC3

w312[3,9] <-rho *sqrt(vC3s[1] *vC3s[3])/nC3

w312[3,10] <-rho *sqrt(vC3s[2] *vC3s[3])/nC3

w312[3,12] <-rho *sqrt(vC3s[4] *vC3s[3])/nC3

w312[4,9] <-rho *sqrt(vC3s[1] *vC3s[4])/nC3

w312[4,10] <-rho *sqrt(vC3s[2] *vC3s[4])/nC3

w312[4,11] <-rho *sqrt(vC3s[3] *vC3s[4])/nC3

## end of 1st arm with 3-arm

w312[5,10] <-rho *sqrt(vC3s[2] *vC3s[1])/nC3

w312[5,11] <-rho *sqrt(vC3s[3] *vC3s[1])/nC3

w312[5,12] <-rho *sqrt(vC3s[4] *vC3s[1])/nC3

w312[6,9] <-rho *sqrt(vC3s[1] *vC3s[2])/nC3

w312[6,11] <-rho *sqrt(vC3s[3] *vC3s[2])/nC3

w312[6,12] <-rho *sqrt(vC3s[4] *vC3s[2])/nC3

w312[7,9] <-rho *sqrt(vC3s[1] *vC3s[3])/nC3

w312[7,10] <-rho *sqrt(vC3s[2] *vC3s[3])/nC3

w312[7,12] <-rho *sqrt(vC3s[4] *vC3s[3])/nC3

w312[8,9] <-rho * sqrt(vC3s[1] *vC3s[4])/nC3

w312[8,10] <-rho *sqrt(vC3s[2] *vC3s[4])/nC3

w312[8,11 ] <- rho *sqrt(vC3s[3] *vC3s[4])/nC3

## end of 2nd arm with 3-arm

dev.total<-sum(dev[])

}

**Initials**

**init 1**

list(mu=-1,sig=.2, beta.m=c(NA, 1,1,1, 1,1,1, 1,1),

delta=c(-1,-1,-1,-1,-1, -1,-1,-1,-1,-1, -1,-1,-1,-1,-1),

a=structure(.Data=c(.1,.1,.1,.1,.1,.1, .1,.1,.1,.1,.1,.1),.Dim=c(6,2)),

aa=c(.1,.1,.1), tau.m1=25) )

**init 2**

list(mu=-.7,sig=.1, beta.m=c(NA, .5, .5, .5, .5, .5, .5, .5, .5),

delta=c(-.7,-.7,-.7,-.7,-.7, -.7,-.7,-.7,-.7,-.7, -.7,-.7,-.7,-.7,-.7),

a=structure(.Data=c(.05,.15, .05, 1.5, .05,1.5, .05,1.5,.05,1.5,.05,1.5),.Dim=c(6,2)),

aa=c(.05,.05,.05), tau.m1=15) )

**init 3**

list(mu=-.5, sig=.5, beta.m=c(NA, .7, .7, .7, .7, .7,.7, .7,.7),

delta=c(-1.5,-.5,-1.5,-.5,-1.5, -.5, -1.5, -.5, -1.5, -.5, -1.5,-.5,-1.5,-.5,-1.5 ),

a=structure(.Data=c(.1,.1,.1,.1,.1,.1, .01,.01,.01,.01,.01,.01),.Dim=c(6,2)),

aa=c(.01,.01,.01), tau.m1=5) )

**init 4**

list(mu=-1.2,sig=1.0, beta.m=c(NA, 1.5,1.5,1.5, 1.5,1.5,1.5, 1.5,1.5),

delta=c(.2,-2.0,.2,-2.0,.2, -2.0, .2,-2.0,.2,-2.0, .2,-2.0,.2,-2.0,.2), ),

a=structure(.Data=c(-.1,-.1,-.1,-.1,-.1,-.1, .1,.1,.1,.1,.1,.1 ),.Dim=c(6,2)),

aaa=(-.1,-.1,-.1), tau.m1=2))

**Social anxiety data: 22 trials**

t[1:15,1:6] treatment indicators, 15 2-arm trials

t2[1:6,1:5] 6 3-arm trials

t3[1:4] 1 4-arm trial

SD[1:21,1:6] pooled SD at follow-up

nC[1:15], nC2[1:6], nC3, nT[1:15], nT2[1:6,1:2] nT3[1:3] sample sizes control and treatment arms

vC[1:15,1:6], vC2[1:6,1:5], vC3 variances of control group

vT[1:15,1:6], vT2[1:6,1:10], vT3 variances of treated group(s)

d[1:15,1:6], d2[1:6,1:10], d3 trial-specific treatment effects relative to placebo, 2-, 3-, 4-arm trials

t[,1] t[,2] t[,3] t[,4] t[,5] t[,6] nC[] nT[] d[,1] d[,2] d[,3] d[,4] d[,5] d[,6]

1 NA NA NA NA NA 176 177 -6.6 NA NA NA NA NA # kasper 2005

1 8 NA NA NA NA 89 176 -7.2 -1.2 NA NA NA NA # asakura 2007

1 2 8 NA NA NA 126 121 -13.8 -0.5 -2.3 NA NA NA # davidson 2004

1 2 8 NA NA NA 148 146 -8.8 -0.5 -2 NA NA NA # westenberg 2004

1 2 9 NA NA NA 138 133 -8.0 -0.4 -3.1 NA NA NA # liebowitz 2005a

1 2 9 NA NA NA 135 126 -8.3 -0.3 -4.7 NA NA NA # rickels 2004

1 6 8 NA NA NA 92 90 -21.4 -4.8 -2.6 NA NA NA # stein 1998

1 3 9 NA NA NA 44 42 -14.2 -7.2 -6.7 NA NA NA # stein 1999

1 2 3 NA NA NA 196 205 -11.9 -0.4 -5.2 NA NA NA # liebowitz 2003

1 3 4 NA NA NA 48 44 -24.8 -11.4 -6.0 NA NA NA # allgulander 1999

1 3 5 NA NA NA 30 30 1.03 0.4 -0.54 NA NA NA # kobak 2002

1 2 6 NA NA NA 151 139 -9.3 -0.7 -3.3 NA NA NA # baldwin 1999

1 2 8 NA NA NA 78 74 -12.64 -0.5 -2.3 NA NA NA # pfizer 2007

1 2 6 8 NA NA 185 184 -13.3 -0.7 -2.5 -2.8 NA NA # lepola 2004

2 3 4 5 6 7 69 134 -0.69 -7.81 -3.52 -5.19 -3.26 -23.3 # van ameringen 2001

END

vC[,1] vC[,2] vC[,3] vC[,4] vC[,5] vC[,6] vT[,1] vT[,2] vT[,3] vT[,4] vT[,5] vT[,6]

882.090 NA NA NA NA NA 942.490 NA NA NA NA NA

286.216 32.040 NA NA NA NA 286.216 28.000 NA NA NA NA

322.560 1.260 45.360 NA NA NA 817.960 1.210 77.440 NA NA NA

852.480 1.480 53.280 NA NA NA 1064.340 1.460 71.540 NA NA NA

680.119 1.380 188.908 NA NA NA 655.477 1.330 182.064 NA NA NA

957.362 1.785 260.458 NA NA NA 914.462 1.666 246.960 NA NA NA

335.800 58.880 20.240 NA NA NA 352.800 57.600 19.800 NA NA NA

376.360 114.490 259.210 NA NA NA 515.290 182.250 213.160 NA NA NA

772.840 1.440 210.250 NA NA NA 789.610 1.440 210.250 NA NA NA

212.116 174.763 14.509 NA NA NA 809.282 156.677 46.615 NA NA NA

576.480 148.109 52.418 NA NA NA 737.666 121.661 68.558 NA NA NA

1100.790 1.510 73.990 NA NA NA 1089.760 1.390 68.110 NA NA NA

688.030 1.000 36.000 NA NA NA 679.387 1.440 60.840 NA NA NA

320.050 1.184 50.024 8.290 NA NA 318.320 1.178 49.754 13.745 NA NA

0.725 156.193 32.911 37.900 35.362 798.201 0.725 156.193 32.911 37.900 35.362 798.201

END

**Three-arm trials**

t2[,1] t2[,2] t2[,3] t2[,4] t2[,5] nC2[] nT2[,1] nT2[,2] vC2[,1] vC2[,2] vC2[,3] vC2[,4] vC2[,5]

1 NA NA NA NA 134 131 130 1271.1776 NA NA NA NA

1 2 NA NA NA 144 133 136 878.5296 0.6808 NA NA NA

1 2 NA NA NA 130 132 133 398.1250 0.6561 NA NA NA

1 9 NA NA NA 132 129 128 760.3200 199.7028 NA NA NA

2 3 7 NA NA 36 39 42 1.6900 182.2500 784.000 NA NA

2 3 4 5 8 92 98 95 2.0700 87.5564 69.6348 69.6348 21.316

END

d2[,1] d2[,2] d2[,3] d2[,4] d2[,5] d2[,6] d2[,7] d2[,8] d2[,9] d2[,10]

-14.6 NA NA NA NA -14.1 NA NA NA NA # stein 2005

-12.8 -0.56 NA NA NA -17.0 -0.73 NA NA NA # liebowitz 2005b

-7.2 -0.2 NA NA NA -6.1 -0.2 NA NA NA # gsk 2006

-16.9 -8.7 NA NA NA -16.3 -7.7 NA NA NA # allgulander 2004

-0.6 -5.9 -25.5 NA NA -0.6 -5.1 -18.7 NA NA # davidson 2004b

-0.59 -4.99 -2.11 -4.88 -4.86 -0.39 -2.23 -0.93 -4.35 -3.28 # blomhoff 2001

END

vT2[,1] vT2[,2] vT2[,3] vT2[,4] vT2[,5] vT2[,6] vT2[,7] vT2[,8] vT2[,9] vT2[,10]

1308.1136 NA NA NA NA 1209.325 NA NA NA NA

926.9568 0.680815 NA NA NA 905.2704 0.688581 NA NA NA

399.6432 0.8281 NA NA NA 398.0557 0.7744 NA NA NA

712.4025 185.76 NA NA NA 774.6048 203.2128 NA NA NA

1.44 174.24 1383.84 NA NA 1.44 158.76 992.25 NA NA

2.205 90.1012 74.1762 75.8912 22.012 2.1375 84.949 68.6375 71.9055 22.012

END

**Four-arm trials**

list(t3=c(1,2,6,8), nC3=95, nT3=c(97, 95, 97), # liebowtz 2002

d3=c(-9.9, -0.5, -2.2, -1.2, -5.3, -0.5, -2.3, -1.3, -6.6, -0.5, -2.7, -1.4),

vC3=c(967.21, 1.5376, 82.81, 25.37),

vT3=c(870.25, 1.5129, 73.96, 22.5, 918.09,1.4884,77.44, 23.66, 900.0,1.5376,75.69,22.5),

# SCALE

#S=c(1, 0.049, 0.5, 0.21, 0.28, 0.19, 1.33, 0.21, 0.47 )) # analyses without standardisation

S=c(1,1,1,1,1, 1,1,1,1)) # analyses WITH standardisation

**Pooled SD at Follow-up**

SD[,1] SD[,2] SD[,3] SD[,4] SD[,5] SD[,6]

30.20556 NA NA NA NA NA # ka

16.91791 5.41821 NA NA NA NA # as

28.66976 1.11154 7.81490 NA NA NA # da

32.64646 1.20825 8.46552 NA NA NA # we

25.84622 1.16424 13.62166 NA NA NA # li

30.60486 1.31451 15.93561 NA NA NA # ri

18.55278 7.63198 4.47464 NA NA NA # st

22.77274 13.55962 15.38614 NA NA NA # st

27.95379 1.20000 14.50000 NA NA NA # li

28.67515 12.50135 6.87841 NA NA NA # al

25.63343 11.61400 7.77740 NA NA NA #ko

33.01039 1.17864 8.25049 NA NA NA # ba

26.06286 1.20241 7.82092 NA NA NA #pf

17.84126 1.08516 7.05352 3.71151 NA NA # le

0.85127 12.49770 5.73685 6.15632 5.94659 28.25246 # va

35.53973 NA NA NA NA NA # st

30.04872 0.82666 NA NA NA NA # li

19.96520 0.86799 NA NA NA NA # gs

27.37024 14.00836 NA NA NA NA # al

1.23157 13.08174 32.53988 NA NA NA #da

1.46251 9.35749 8.41810 8.51726 4.6674 NA # bl

30.22556 1.23255 8.80040 4.84736 NA NA # li

END

For analyses without standardization the data structure SD[,] is not entered, and the SD[,] are set to 1 by adding the following to the code: for (i in 1:22) { for (j in 1:6) { SD[i,j] <- 1 } } .

Models with Fixed mapping ratios are generated by removing variables by setting sbeta[i,k] <- sbeta.m[k]. Mappings can be fixed at 1 by setting sbeta[i,k] <- 1.
